# Machine learning in the analysis of mental health at work: a scoping review

**DOI:** 10.1093/joccuh/uiag014

**Published:** 2026-03-09

**Authors:** Pekka Varje, Ari Väänänen, Olli Haavisto, Ilkka Kivimäki, Simo Taimela, Tiina Kalliomäki-Levanto

**Affiliations:** Finnish Institute of Occupational Health, Work Ability and Work Careers, P.O. Box 40, FI-00032 Työterveyslaitos, Helsinki, Finland; Finnish Institute of Occupational Health, Work Ability and Work Careers, P.O. Box 40, FI-00032 Työterveyslaitos, Helsinki, Finland; Finnish Institution of Occupational Health, ICT and Digital Services, Helsinki, Finland; Finnish Institution of Occupational Health, ICT and Digital Services, Helsinki, Finland; Terveystalo Plc, Medical Leadership, Helsinki, Finland; Finnish Institute of Occupational Health, Work Ability and Work Careers, P.O. Box 40, FI-00032 Työterveyslaitos, Helsinki, Finland

**Keywords:** artificial intelligence, literature review, mental disorders, natural language processing, occupational health, work life

## Abstract

**Objectives:**

This scoping review aimed to assess the role of machine learning in workplace mental health research by systematically analyzing existing studies to understand current methodologies, applications, and trends.

**Methods:**

We conducted a comprehensive search across multiple databases, including EBSCO, Scopus, ProQuest, Web of Science, PsycINFO, IEEE, and ACM, screening a total of 5600 abstracts. Altogether, we analyzed 92 journal articles, conference papers, and book chapters published before September 2025.

**Results:**

Since 2020, there has been a notable increase in publications on the topic. Studies have mainly employed cross-sectional designs (73%) and workplace questionnaires (51%) targeting specific occupational groups (67%), particularly from Asia excluding China (41%). Supervised learning methods, such as Random Forest and Neural Networks, have been frequently utilized to investigate conditions like depression, burnout, and anxiety. Most studies predicting mental health at work using machine learning are currently conducted by data scientists as single-measurement studies, whereas longitudinal studies from medicine, epidemiology, social sciences, or behavioral sciences are comparatively rare. In the context of machine learning, prediction denotes the model's ability to infer outcomes based on input data. However, most publications do not systematically analyze the temporal dynamics of mental health or forecast mental health outcomes from an epidemiological perspective.

**Conclusions:**

The application of machine learning in occupational mental health research remains in its preliminary stages, with a primary focus on methodology and computer science. The review highlights the necessity for interdisciplinary collaboration to fully leverage the potential of machine learning in advancing occupational health research.

## Teaser

**Key points**
 

- **What is already known on this topic**: Work-related factors are key determinants of mental health. Most existing research relies on conventional statistical analyses of survey-based datasets. Machine learning has emerged as a promising approach for predicting mental health risks and informing data-driven interventions.
- **What this study adds**: Applications of machine learning in work-related mental health research remain at an early stage. Although publications have increased rapidly, most emphasize algorithmic performance rather than robust study design or theory-driven inquiry. Current research is concentrated in Asia, primarily uses questionnaire data, and focuses on predicting depression and burnout through supervised learning. Large-scale, longitudinal, and multimodal approaches are uncommon, limiting clinical and occupational relevance. This review highlights critical gaps and opportunities for advancing this interdisciplinary field.
- **How this study might affect research, practice, or policy**: This review calls for stronger collaboration between data scientists and occupational health researchers. Integrating diverse data sources and adopting longitudinal designs could enhance predictive accuracy and enable early detection of mental health risks. Such advances may inform workplace policies that support ethically governed and voluntary mental health screening and tailored interventions.

## 1. Introduction

Mental health problems pose a significant burden on work organizations and societies.^1^^,^^2^ They are among the leading causes of work disability and sickness absence worldwide, contributing substantially to productivity loss and economic costs.^3^ Enhancing our understanding and the prevention of mental disorders is essential for promoting social and economic sustainability.^4^

Work is widely recognized as a major contributor to mental health, yet most existing scientific research on occupational mental health and its social and psychological factors relies on traditional statistical methods applied to survey data and pre-structured datasets.^5^ However, the rapid advancement of digital networking, data storage, and data collection capabilities is expanding access to new types of datasets, necessitating novel methodological approaches.^6^

Machine learning methods hold significant promise as a cost-effective, high-impact tool for enhancing our understanding, support, and prevention of mental disorders.^7^ Integrating computational power into health research could lead to substantial advancements in analyzing disease etiology, prediction, early detection, and management.^8^ For instance, Grzadzielewska^9^ suggests that machine learning techniques could be employed to develop new theoretical models of burnout and data-driven interventions, including automated supervision processes that improve working conditions and mitigate burnout among workers. Consequently, machine learning has the potential to drive socioeconomic change and address persistent issues related to the costs of occupational distress and ill health.

However, despite several promising examples, the application of machine learning in mental health research remains in its preliminary stages. For instance, Hashmi and Yadav's^10^ systematic literature review on machine learning in stress research conducted in 2019 revealed that the use of these technologies for measuring occupational stress is still an emerging field, with the existing literature constrained by numerous limitations. Furthermore, few practical solutions have been developed, and the potential of machine learning to leverage new big-data sources remains underutilized.^11^

In this review article, we provide an overall picture of the research literature on machine learning–based analysis of mental health in the workplace. Our aim was to assess the status and scope of occupational research where mental health or the risk of mental health deterioration is predicted using machine learning methods, and to elucidate the role of data sciences in advancing this area of study. In the scoping review, we explored the types of studies utilizing machine learning methods that have been conducted in the area of explaining work-related mental health. The specific research questions were: (1) What scientific fields and approaches are prominent in this area of research? (2) In which parts of the world have these studies primarily been conducted and with what methods? (3) What have been the study populations and research designs? (4) What areas for development can be identified?

### 1.1 Categories for literature review

This review employs a specific thematic scope and classification system. We examined studies that utilize machine learning in the context of mental health and workplace mental health issues. Consequently, we included studies addressing both clinical conditions, such as depression and anxiety disorders,^12^ and subjective measures of mental health, such as job burnout symptoms.^13^ We also evaluated the medical applicability of the findings.

A key characteristic of machine learning is its ability to perform classification tasks on research material and generate predictive models based on large datasets. Machine learning–based classification models have demonstrated proficiency in identifying diseases and categorizing patients based on symptom descriptions in medical records.^14^^,^^15^ Furthermore, recent text mining methods and natural language processing (NLP) facilitate the analysis of extensive databases containing natural language.^16^ NLP techniques can extract dimensions such as symptom descriptions or emotional states from free-form text, analyzing them as though they were structured data. This capability allows researchers to utilize novel materials for classification or prediction purposes.^17–21^ Consequently, it is crucial to identify the study designs and datasets employed in the analyses.

In data science, it is widespread practice to employ multiple analytical methods on the same dataset, allowing researchers to compare the results to assess variations among models and identify the most effective ones. This approach not only enhances the robustness of findings but also ensures that the chosen model is well suited to the dataset's characteristics. In our review, we systematically extracted the various methods employed in the publications. This provides insights into prevailing trends in methodological preferences and highlights which techniques are currently considered most reliable in the field.

## 2. Methods

### 2.1 Type of review

Given that machine learning is a novel and rapidly evolving methodological approach in mental health research, our focus was on exploring the scope of the research, mapping key concepts, types of evidence, and identifying gaps. As suggested by Arksey and O’Malley,^22^ the primary purpose of a scoping review is to examine the extent, range, and nature of research activity within a specific field. In conducting this review, we followed the Joanna Briggs Institute's manual for the scoping review framework.^23^ In reporting this scoping review, we adhered to the PRISMA-ScR guidelines.^24^

### 2.2 Search strategy

Our search strategy was guided by 3 inclusion criteria for the publications analyzed: (1) the studies must employ methods related to machine learning, (2) the outcomes must pertain to mental disorders or other subjective measures of mental health, and (3) the publications must be relevant to a work-life context, with the study population comprising workers. The search terms included keywords associated with mental health, employees or working populations, and machine learning. Specific search terms are detailed in Table S1. We did not set limits on the publication date. The initial searches were conducted across multiple databases, including EBSCOhost, Scopus, ProQuest, Web of Science Core Collection, APA PsycINFO, IEEE Xplore, and ACM Digital Library, in June 2023, and the searches were updated using an identical search strategy in September 2025.

### 2.3 Study selection

After deduplication, all remaining records were independently screened by 2 reviewers at both the title-and-abstract stage and the full-text stage. Discrepancies in the title-and-abstract screening were resolved by a third reviewer, while disagreements during the full-text review were resolved through consensus among all authors.

During the screening process, studies were excluded based on the following criteria: (1) Wrong study focus: machine learning or artificial intelligence was used as an intervention, support tool, or digital therapeutic component rather than as an analytic method for examining mental-health-related data. (2) Wrong outcome: the outcome did not pertain to mental health, or the mental health–related outcome was insufficiently defined; studies focusing solely on work stress were excluded unless work stress was examined alongside a mental health–related variable. (3) Wrong population: the study population did not represent employees, workers, or a work-life context. (4) Dataset not disclosed: the publication did not report the data source or the type of data. (5) The publication was not written in English. (6) The full text was not available. (7) The publication was not published in the format of a scientific report.

In the study selection process, machine learning was defined as computational approaches that learn patterns from data and improve performance on a task through algorithmic training, enabling models to generalize beyond the data used for model development. Eligible methods included supervised and unsupervised algorithms such as Random Forest, Gradient Boosting, Support Vector Machines, Neural Networks, and clustering approaches. Logistic regression was included only when the authors explicitly defined or used it as a machine learning method—typically in predictive model training, validation, or algorithm comparison—and excluded when used solely as a traditional inferential statistical model. Logistic regression is classified as a supervised learning algorithm in several machine learning–oriented publications,^25^^,^^26^ whereas statistical literature commonly treats it as an inferential model,^27^ reflecting its context-dependent role.

### 2.4 Data extraction and analysis

For each publication included in the review, data were extracted by 2 reviewers using a standardized data extraction template, with disagreements resolved by consensus. The template covered items related to background information, aims, methods, data, study population, results, and applicability; the full template is available in Table S2.

Most variables extracted during the data extraction phase—such as publication year, population size, machine learning algorithms, and outcome measures—were directly reported in the included publications. Some variables, however, required additional operational clarification because their definitions were not consistently provided.

The main discipline was determined from the first author’s institutional affiliation and department, cross-checked against the affiliations of other authors and the journal’s stated scope.

Method type was coded as prediction when the study’s primary aim was to forecast an individual’s future health outcome (eg, disease onset or a clinical event). Classification was used when the study aimed to assign individuals to predefined groups based on existing characteristics (eg, clinical profiles or patient subgroups) without predicting future outcomes. Data mining referred to analyses of large datasets to identify meaningful patterns or relationships; NLP to techniques enabling computers to interpret or generate human language; and clustering to grouping data based on similarity to reveal underlying structure. Coding decisions were guided by the study design and the stated aims as provided by the authors, and ambiguous cases were resolved through team discussion.

The use of supervised or unsupervised methods was occasionally stated explicitly in the included publications, but the classification required interpretation in all cases. Supervised methods were identified by the use of labeled data, whereas unsupervised methods were identified when unlabeled data were used to uncover hidden patterns, groupings, or structures without predefined targets.

Medical application categories (diagnosis, prognosis, epidemiology, treatment) were assigned based on each study’s stated aims and the application context of the modeled outcome. A single study could receive multiple categories when its objectives spanned more than 1 application area; for this reason, the total count of applications exceeds the number of included studies.

Extracted data were analyzed using frequency analysis. The screening and data extraction were conducted with the review tool Covidence (Covidence systematic review software, Veritas Health Innovation, Melbourne, Australia; available at www.covidence.org), which is a web-based collaboration software platform that streamlines the production of systematic and other literature reviews. The frequency analyses were performed using IBM SPSS Statistics version 30.0.0.0 (172).

## 3. Results

### 3.1 Overview of publication characteristics

**Figure 1:**
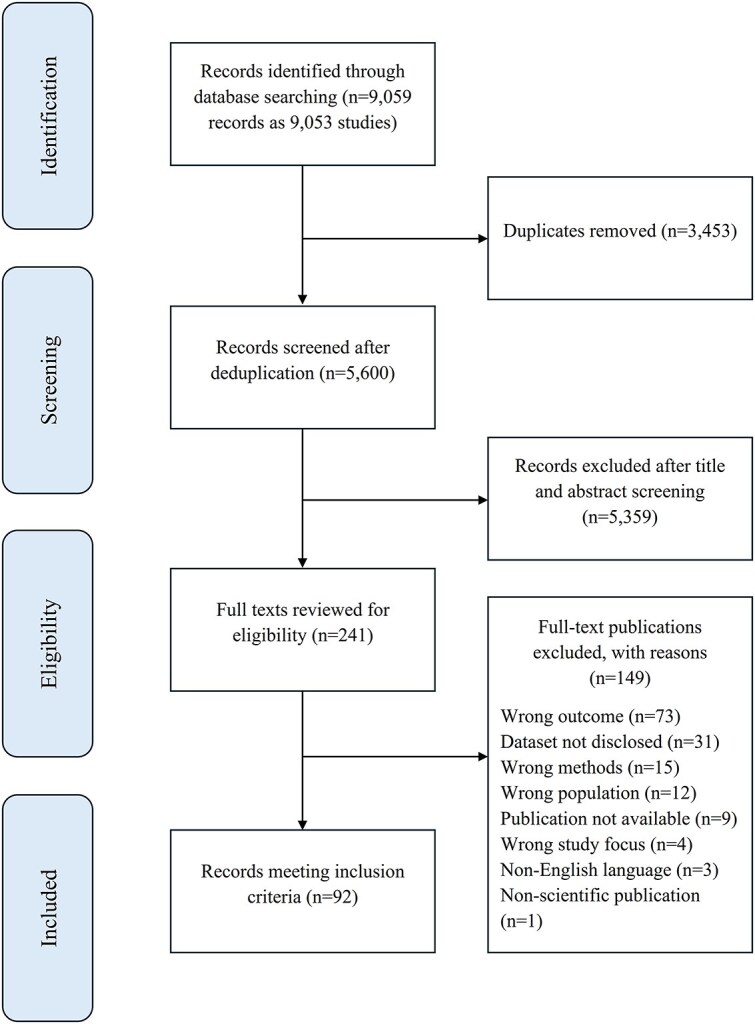
PRISMA flow diagram of the study selection.

**Figure 2:**
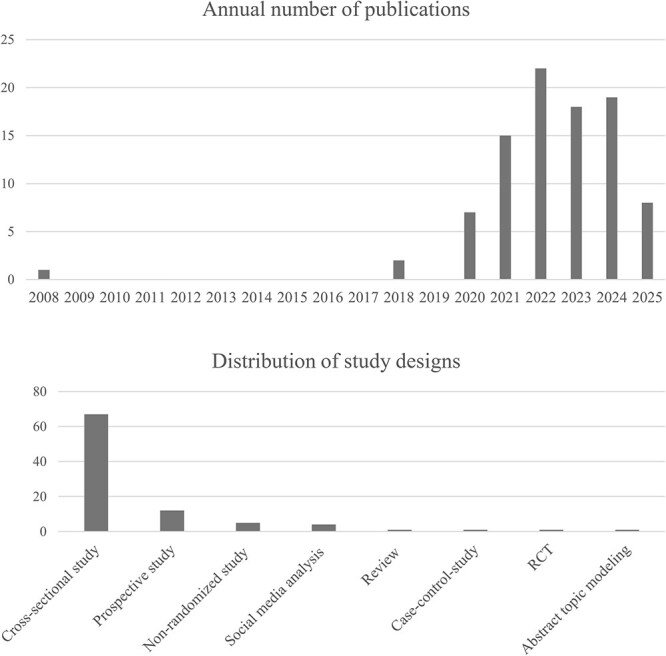
Annual number of publications (2008-2025) and overall distribution of study designs (note that the count for 2025 reflects publications up to August only, which explains the small number of publications).

The database searches identified a total of 9059 references, which Covidence clustered into 9053 studies before deduplication. After 3453 duplicates were automatically removed, 5600 items remained for title and abstract screening. After screening and full-text review, 92 publications met the inclusion criteria, comprising 72 journal articles, 1 chapter in an edited book, and 19 conference papers. A detailed account of the screening process is provided in Figure 1. The included publications were published between 2008 and 2025, with only 3 appearing before 2020. The number of publications increased markedly from 2020 onwards, with a total of 89 publications between 2020 and September 2025. On average, the annual publication rate was slightly below 20 publications. Figure 2 illustrates the sharp increase in publications from 2020 onward, together with the overall distribution of study designs used in the included studies.

According to Table 1, approximately 41% of the analyzed studies were conducted in Asia, excluding China, with consideration given to the study populations. A comparable number of studies, totaling 38, were collectively conducted in the USA, Europe, and China, while 14 studies took place in other regions. The reviewed publications included 1 multi-region study and 1 study where the source of the population sample was not reported.

**Table 1:** Regions in which the studies were conducted, based on the study population.

Region	Number of publications (%)^a^
**Asia excluding China**	38 (41)
**Europe**	14 (15)
**China**	12 (13)
**USA**	11 (12)
**The Americas excluding USA**	7 (8)
**Africa**	4 (4)
**Oceania**	3 (3)
**Multi-region**	2 (2)
**Not reported**	1 (1)

^a^Percentages are calculated as the proportion of publications (*n* = 92) conducted in each region.

Among the included publications, 89 were original studies, 2 were research protocols, and 1 was a review. The primary discipline of the publications was computer science, accounting for 41 publications, or almost half of all publications. Twenty-three publications, representing one-fourth of the total, were from the field of medical sciences. Fewer publications originated from epidemiology (16 publications), social sciences (6 publications), and behavioral sciences (6 publications).

### 3.2 Study population and methodology

The analyzed publications utilized a wide range of population sample sizes, with the smallest sample consisting of 42 individuals and the largest comprising 5 831 302 individuals. The average population size was 82 195, while the median size was 871. In 10 publications, the population sample size was not clearly specified. In 2 publications, this question was not applicable: 1 publication was a review, while the other utilized scientific abstracts as study material. Most publications focused on occupational groups (62 publications), and 14 used a general population sample. Six publications based their analyses on samples gathered from work organizations, 5 utilized samples of patients with mental health diagnoses, and 4 employed an unspecified online recruitment sample.

Most of the publications (67 publications) were based on a cross-sectional study design. Only 12 publications utilized a prospective study design, while 5 were nonrandomized experimental studies. The reviewed publications also included 4 social media analyses, 1 case–control study, 1 randomized controlled trial, 1 analysis of scientific publications, and 1 literature review.

The most frequently used dataset in the analyzed studies was a workplace questionnaire (47 publications). Other datasets included online questionnaires (15 publications), open-source surveys (14 publications), sensor data (11 publications), national survey data (6 publications), national register data (5 publications), clinical data (4 publications), social media posts (4 publications), employee registers (2 publications), and scientific literature (2 publications including the review article). A combination of different datasets was used in 19 publications, with the most common pairing being workplace questionnaires with sensor data (10 publications). Nearly all publications relied solely on structured data, with only 8 utilizing unstructured text data.

In most publications (74 publications), the models were designed to predict specific outcomes from the source data, despite the prevalent use of cross-sectional study designs, which lack temporal separation between predictors and outcomes. Fifteen publications concentrated on classifying cases with different labels—a task closely related to prediction. Nine publications employed machine learning for NLP, while 8 publications focused on data mining. Additionally, 6 publications centered on cluster analysis—a method closely related to data mining.

Sixteen publications combined different aims within a single study. Most publications utilized supervised methods, while 10 employed a combination of supervised and unsupervised methods, and 8 relied solely on unsupervised methods.

More publications employed multiple algorithms (57 publications) than those using a single algorithm (33 publications). As shown in Table 2, the Random Forest algorithm was the most popular, followed by various forms of Gradient Boosting, Support Vector Machines, Neural Networks, Decision Trees, and Logistic Regression. In many cases, the purpose of utilizing multiple algorithms was to compare the classification abilities of different models and determine the most suitable model for the prediction task. In 2 reviewed publications, the algorithms were not clearly specified, or the question was not relevant.

**Table 2:** The 10 most common algorithms used in the study designs.

Rank	Algorithm	Number of publications^a^ (%)
**1**	Random Forest	43 (47)
**2**	Gradient Boosting	35 (38)
**3**	Support Vector Machine	31 (34)
**4**	Neural Network	26 (28)
**5**	Decision Tree	23 (25)
**6**	Logistic Regression	20 (22)
**7**	Naïve Bayes	14 (15)
**8**	Ensemble Learning	12 (13)
**9**	K-Nearest Neighbours	9 (10)
**10**	Ridge or Lasso Regression	9 (10)

^a^The number of publications exceeds 92, because a single publication can utilize multiple algorithms. Percentages, however, are calculated as the proportion of publications (*n* = 92) that reported each algorithm.

### 3.3 Outcome, results, and contributions

In most publications, the focus was on analyzing the data to detect individuals who may have mental health problems. Only 4 publications focused on individuals with previously diagnosed mental disorders, analyzing their employment status, sick leave, or welfare dependency. The outcomes were predominantly self-reported, with only 7 publications utilizing clinical or register-based data.

As indicated in Table 3, the most common outcome was related to depression, followed by burnout, anxiety, and unspecified mental disorders. It was common for reviewed publications to include multiple outcomes within a single study (26 publications).

**Table 3:** The mental health–related outcomes of the reviewed publications.

Outcome	Number of publications^a^ (%)
**Depression**	33 (36)
**Burnout**	33 (36)
**Anxiety**	16 (17)
**Unspecified mental disorders**	14 (15)
**Work stress (alongside a mental health–related outcome)**	8 (9)
**Psychological distress**	6 (7)
**Suicidal ideation**	5 (5)
**Common mental disorders**	5 (5)
**Sleep disorder**	3 (3)
**Post-traumatic stress disorder**	2 (2)
**Affective disorders**	2 (2)
**Compassion fatigue**	1 (1)

^a^The number of publications exceeds 92, because the same publication can contain multiple outcomes. Percentages, however, are calculated as the proportion of publications (*n* = 92) that reported each outcome.

We classified the publications into 5 categories, assessing their primary usability or main application target. Primary contributions of these studies were in the field of epidemiology (65 publications), with some applicability to treatment (21 publications) and diagnosis (15 publications). Only very few of the reviewed studies provided contributions to medical prognosis (4 publications). For 3 publications, we did not identify clear applicability for any specific target.

Table 4 presents a comprehensive overview of the aims and key findings from the reviewed publications. These studies primarily focused on identifying factors affecting mental health, burnout, and emotional well-being across various professional settings. Utilizing machine learning models, they predicted outcomes such as burnout, depression, anxiety, and post-traumatic stress disorder (PTSD) by analyzing data from heart rate variability, sociodemographic factors, electronic health records, and social media content.

**Table 4:** The aims and the main results of the included publications.

Publication	Aim of study	Main results
**Adamapoulos et al, 2025^28^**	Forecast work-related burnout risks with machine learning	The study reported that machine learning methods may offer new opportunities for mitigating burnout risks
**Adapa et al, 2022^29^**	Identify key characteristics influencing burnout risk among health professionals during COVID-19	The authors identified factors linked to burnout symptoms with 2 items, but its cross-sectional nature limited contributions to mental health research
**Adeniji et al, 2022^30^**	Develop a machine learning model to predict mental health disorders	The authors noted that their model may assist mental health professionals in early diagnosis, although they did not evaluate its practical usefulness
**Adorjan et al, 2024^31^**	Create a simple model for health care workers to self-assess pandemic-related psychological distress	According to the results, a 10-variable model predicted psychological distress risk, potentially aiding health care workers in monitoring personal and team-based risks
**Agarwal et al, 2023^32^**	Analyze social media content from emergency medicine physicians to assess emotional well-being changes during COVID-19	The authors argue that social media content may reveal thematic shifts in language related to anxiety, depression, and loneliness, offering a new real-time evaluation of health care workers' mental health
**Alayo et al, 2025^33^**	Develop a machine learning model to predict future suicidal thoughts and behaviors	According to the study, machine learning models offer high discrimination and classification capacity, enabling early detection of mental health risks in health care workers
**Atachagua et al, 2022^34^**	Identify burnout syndrome in frontline health and social care workers using AI	The study reported that Neural Network models were able to identify burnout states among frontline health and social care workers
**Awal & Rao 2021^35^**	Analyze mental illness survey data to explore effects on career prospects	The authors suggested that a Decision Tree model was suitable, although the reporting of the main results remained unclear
**Bakkeli 2023^36^**	Predict depression risk, assess socioeconomic status roles, and select best models for each COVID-19 phase	The study found that different machine learning models varied in their net benefits; Decision Trees and Regularized Regression models performed best in identifying depression, with social factors emerging as key explanatory variables
**Baniadamdizaj & Baniadamdizaj 2023^37^**	Develop a predictive tool for accurately forecasting teacher burnout	The authors concluded that the machine learning algorithms used in their study effectively predicted teacher burnout
**Bh et al, 2022^38^**	Identify risk factors for mental illness among employees	The study found that XGBoost performed best in early detection tasks, and the authors recommended exploring hybrid techniques
**Bjerregaard 2023^39^**	Predict welfare dependency among individuals initially on long-term sickness absence due to mental disorders	The study reported that machine learning models such as Extreme Gradient Boosting supplemented traditional approaches like multinomial logistic regression by enabling benchmarking of predictive performance and highlighting relevant associations
**Byeon 2024^40^**	Identify factors influencing suicidal ideation in workers with mental disabilities	The authors reported that machine learning models highlighted the importance of psychological and social factors in predicting suicidal ideation among workers with mental disabilities
**Cascella et al, 2025^41^**	Develop and evaluate dense Neural Network models to predict burnout from occupational, psychological, and behavioral factors	The study found that the high-risk burnout group showed elevated anxiety and depression levels; the authors also discussed how AI-based models may support improved identification of burnout risk
**Diez-Pinol et al, 2008^42^**	Evaluate linkages between personal, organizational, and cultural variables affecting burnout and vigor	The study reported that its model explained 59% of the variance and identified both high-burnout-risk and enhanced health profiles, which the authors noted could help doctors and hospitals better understand patterns of burnout and vigor
**Doki et al, 2021^43^**	Predict psychological distress using sociodemographic, lifestyle, and sleep factors, comparing AI models with psychiatrists	The study reported that the AI model effectively screened workers for psychological distress, outperforming psychiatrists in predicting severe—but not moderate—distress
**Fernandez et al, 2018^44^**	Develop and validate a risk algorithm predicting common mental disorders at 12 months in workers	The study demonstrated that an algorithm could be developed to identify both overall and modifiable risks of common mental disorders among working men, achieving good discriminatory performance according to the authors’ evaluation
**Fukuda et al, 2020^45^**	Build a binary model to predict depression, positive, and anxiety moods	The study reported estimated F1-scores of 0.776 for depression, 0.610 for positive mood, and 0.756 for anxiety
**Gamage & Asanka 2022^46^**	Propose a screening system to predict mental distress using machine learning, identifying high-risk employees for early assistance	The authors suggested that machine learning could automate aspects of mental disorder screening and support earlier detection compared with manual methods
**Geoffrion et al, 2023^47^**	Identify health care workers at risk of anxiety, depression, and PTSD	The authors reported that their proof-of-concept model could screen for psychological distress using fewer questions, potentially reducing monitoring burden
**Grządzielewska 2021^9^**	Present insights into machine learning methods for burnout prediction tasks	The review suggested that machine learning approaches may support the development of new theoretical models of burnout and data-driven interventions
**Guo et al, 2024^48^**	Examine job crafting and leisure crafting's impact on burnout using machine learning models	The study found that job crafting was the top predictor of burnout, followed by leisure crafting in Random Forest and Gradient Boosting models
**Gupta et al, 2024^49^**	Evaluate rajyoga meditation versus stress management counseling in addressing burnout and automating ECG-based predictions with machine learning	The study reported that rajyoga meditation led to a greater reduction in burnout compared with stress-management counseling, and that their machine-learning model was able to identify burnout from ECG data effectively
**Gupta et al, 2021^50^**	Evaluate burnout prevalence and develop an HRV-based ML model to detect burnout in HCWs during COVID-19	According to the results, burnout rates were higher among second-line workers at 20.5% compared with frontline at 14.9%. Key burnout factors were identified as stress, job dissatisfaction, chaotic environments, and COVID-19's impact on mental wellbeing
**Gupta et al, 2021^51^**	Estimate burnout prevalence in HCWs during COVID-19 using Mini Z-scale and develop AI models for detection	The authors argue that ECG data from those with burnout can be used to develop AI models predicting stress and burnout in health care workers during the COVID-19 era
**Havaei et al, 2021^52^**	Identify key work environment predictors of nurse mental health	The authors argue that routine assessments of nurses' work environment and mental health are crucial predictors of mental health, followed by work–life balance, psychological protection, and workload management
**Henry & Isa 2022^53^**	Predict mental health treatment needs for IT employees	The authors identified key features associated with IT employees’ mental health–related treatment needs
**Hernandez et al, 2024^54^**	Develop models for burnout prediction and classification using machine learning	According to the results, Linear Discriminant analysis, Decision Tree, and Quadratic Discriminant Analysis excelled in model performance, predicting burnout with 95.8% accuracy
**Hoque et al, 2022^55^**	Assess the potential of social media to evaluate workers' emotions toward the workplace	The study reported that work-related sentiment patterns on social media could help identify opportunities to support workers’ well-being
**Hossain et al, 2024^56^**	Classify workplace mental health features using machine learning	The study reported that Support Vector Machine, Neural Network, Extreme Gradient Boosting, and Random Forest models accurately classified mental health–related discussions, with the Neural Network achieving the highest accuracy (93.98%), and that these models provided insights that could potentially support targeted well-being initiatives
**Imanthika et al, 2023^57^**	Build a machine learning model to predict employee mental health and treatment needs	According to the results, an ensemble algorithm merging 5 models yielded best accuracy, precision, recall, f-measure, and error values
**Irfan et al, 2023^58^**	Evaluate COVID-19's psychological effects on Saudi Arabian health care professionals	The study concluded that Saudi frontline health care workers experienced notable levels of anxiety and depression during COVID-19
**Jalandra et al, 2022^59^**	Explore factors related to stress, anxiety, and depression among HCWs during a pandemic	According to the results, severe stress was present in 17% of respondents; the model achieved an *R*^2^ of 0.28. Major stress factors included personal time scarcity, age, duty hours, marital status, and being a resident physician
**Jamalirad & Jajroudi 2023^60^**	Predict tech industry employees' perception of mental health support and ascertain related factors	The authors argue that machine learning models are reasonably accurate and support predicting employee mental health in the technology industry
**Katarya & Maan 2020^61^**	Find features influencing employee mental health	According to the results, a Decision Tree classifier showed the best performance, especially in precision
**Kawakami et al, 2021^62^**	Describe a research protocol for a new iCBT stress management program using AI to improve depression during COVID-19	Results not available
**Kim et al, 2023^63^**	Build models to detect factors associated with workplace depression	The study argues that machine learning models could effectively predict depression risk, identifying work-related stress, burnout, social problem-solving, and meaning at work as risks
**Kumari et al, 2024^64^**	Develop models for perceived mental health and life stress	Perceived health, age group, and mental health compared with pre-pandemic were identified as top predictors for perceived stress, with pre-pandemic mental health being the most significant for life stress
**Kurisu et al, 2023^65^**	Develop action plans for health promotion based on sick leave predictions at a Japanese manufacturing plant	The model achieved an area under the curve of 0.882; job stress, younger age, and certain departments were identified as predictors of sick leave due to mental disorders
**Kutsuna et al, 2022^66^**	Use NLP to score medical records and clarify the relationship between sick leave duration and emotional word use	According to the study, professional assessments and natural language processing of medical records can predict the timing for return to work
**Li et al, 2024^67^**	Identify depression risk factors among nonmanual workers in China	Age, fatigue, sleep quality, overeating, waist-to-hip ratio, and cholesterol were identified as depression risk factors in white-collar workers
**Li et al, 2021^68^**	Identify core burnout factors from individual, job-related, organizational, and social aspects	The authors argue that an association rule analysis identified occupational hazards related to burnout among construction managers
**Li et al, 2024^69^**	Compare tweets from younger and older construction workers and nurses to assess mental health risks from work safety issues	According to the results, nurses' tweet sentiment was more positive than construction workers’, but this changed during COVID-19
**Lou et al, 2022^70^**	Investigate machine learning's ability to identify burnout using electronic health records	The study reported that including baseline burnout scores improved the model’s performance, even though the baseline scores themselves did not differ significantly
**Lu et al, 2024^71^**	Identify and rank mental health determinants in Chinese physicians and nurses, comparing impacts	According to the results, anxiety affected 31.0% of physicians and 53.3% of nurses; depression affected 30.8% of physicians and 47.9% of nurses. Anxiety was linked to cynicism and exhaustion in physicians, while exhaustion impacted depression in nurses
**Mahajan et al, 2021^72^**	Determine health care worker stress using electrocardiograms during COVID-19	The authors suggest that stress among employees is reflected in ECG signal changes captured by machine learning classifiers
**Majcherek et al, 2022^73^**	Evaluate and rank the importance of mental health determinants: lifestyle, demographics, and socioeconomic status	The study argues that European employees' mental health is strongly influenced by BMI, age, and social support
**Malgaroli et al, 2023^74^**	Compare treatment concerns for health care workers with matched non-HCW patients using psychotherapy transcripts from telemedicine during COVID-19	According to the results, treatment topics linked to anxiety and depression included hospital unit work, mood disturbances, and sleep issues
**Mallick & Panda 2024^75^**	Assess the severity of mental health issues among tech industry employees	According to the results, Random Forest achieved 79% accuracy, 77% recall, and 77% precision in predicting mental health issues among tech professionals
**Martin et al, 2023^76^**	Identify personal and professional characteristics of nurses with increased burnout and stress	The study reported that during the COVID-19 pandemic, 62% of the sample reported increased workloads, with high proportions feeling emotionally drained, fatigued, burned out, or at their limit regularly
**Megala et al, 2024^77^**	Improve precision in workforce depression identification	The authors reported that their model achieved 97% accuracy in detecting subtle indicators of employee depression
**Minelli et al, 2022^78^**	Investigate psychological distress in MHWs post-lockdown in Italy, assessing COVID-19, sociodemographic, and professional impacts and previous trauma	The study suggests that mental health workers faced increased distress during COVID-19, especially nurses, highlighting the need for targeted interventions
**Nam et al, 2021^79^**	Identify depression-associated factors using XGBoost from survey data and understand multifactorial features with Network Analysis	According to the authors, XGBoost and Network Analysis revealed depression-related factors that could be applied in epidemiological studies
**Njoroge et al, 2023^80^**	Identify and validate sensor signatures predicting major depressive episode risk in Kenyan health care workers	The authors suggest that a scalable mobile technology platform can be crucial for understanding and improving mental health outcomes
**Ogasawara et al, 2022^81^**	Evaluate if online lectures developed using text-mining can reduce anxiety among health workers in non-epicenter COVID-19 areas	The study reported that online lectures developed using text-mining methods significantly reduced COVID-19–related anxiety and increased knowledge confidence among health care workers in non-epicenter areas
**Park & Lee 2022^82^**	Examine predictors of suicidal ideation using individual characteristics, emotional states, and work environments	According to the results, machine learning could efficiently predict suicide risk using variables like working environments
**Park & Lee 2022^83^**	Predict suicidal ideation based on shift work using machine learning techniques	The study suggests that suicidal ideation was highest among shift workers experiencing depression and low quality of life, and that machine learning could aid early screening
**Park et al, 2023^84^**	Detect factors contributing to depression among farmers	The study linked physical status, sleep time, and depression, with Category Boosting achieving 79.7% accuracy and 81.4% F1 score, outperforming other ensemble models. Random Forest showed highest sensitivity at 90% and 81.3% F1 score
**Perego et al, 2022^85^**	Identify psychological and personal factors influencing responses to the COVID-19 pandemic	The authors argue that a flexible data-mining approach using Regression Trees for repeated measures identified risk factors and classification rules, potentially aiding targeted interventions for health care workers
**Petit et al, 2025^86^**	Identify key depression predictors among French farmers using XGBoost	According to the results, the top depression predictors were working years, age, sex, experience, job security, income, and health conditions
**Pillai et al, 2024^87^**	Predict key factors contributing to health care provider burnout with machine learning	The study suggests that factors like time pressure, work-life integration, inadequate technology, and moral distress could significantly impact health care provider burnout prediction
**Portugal et al, 2022^88^**	Use machine learning to predict depression and PTSD symptoms from psychometric data	According to the study, professional recognition protects against posttraumatic stress, while social isolation increases vulnerability
**Posada-Quintero et al, 2020^89^**	Understand differences in risk factors and symptoms of burnout among schoolteachers	The study identified satisfaction with income as the top burnout risk factor, followed by overtime work and sanctions for low performance. Fatigue and headaches were identified as key symptoms
**Priya et al, 2020^90^**	Predict anxiety, depression, and stress with machine learning algorithms	The study reported that the Random Forest model achieved the highest accuracy, and that its performance metrics indicated good ability to correctly identify negative cases
**Ravuri et al, 2020^91^**	Design group-specific well-being models using multimodal longitudinal data from health care workers	According to the authors, iterative participant clustering models outperformed baseline systems and nonclustering models
**Ravuri et al, 2020^92^**	Explore Group-Specific Machine Learning models for estimating hospital workers' well-being using longitudinal multimodal data	The study reported that Group-Specific Machine Learning models outperformed general models in estimating well-being
**Rensi et al, 2024^93^**	Detect key burnout topics among 3 occupational groups via topic modeling	The authors observed that burnout abstracts rarely addressed prevention or cultural context and suggested that more comprehensive models could improve understanding
**Renugadevi et al, 2023^94^**	Use machine learning to evaluate workplace mental health and forecast potential employee concerns	According to the results, machine learning techniques provided accurate results, with each classifier exceeding 82% accuracy
**Rocha et al, 2025^95^**	Identify burnout profiles and protective patterns among oncology nurses	The authors identified 6 protective patterns against burnout among oncology nurses, including permanent contracts and supportive work environments
**Rosaline et al, 2022^96^**	Use modified deep learning Neural Networks to assess stress patterns in IT professionals and identify key stress components	The study suggests that gender, family history, and health benefits significantly influence stress
**Rykov et al, 2021^97^**	Examine digital biomarkers from wearable sensor data to detect depression risk in workers	According to the results, digital biomarkers showed limited depression detection ability in the general sample but achieved 80% accuracy in balanced subsamples
**Sánchez 2018^98^**	Examine demographic and rehabilitation variables affecting employment outcomes for people with affective disorders in US vocational rehabilitation	The study suggests that job placement, on-the-job support, and job search assistance may predict successful employment outcomes for individuals with affective disorders
**Santoso et al, 2024^99^**	Identify the most effective model for predicting mental health treatment outcomes	According to the results, Extreme Gradient Boosting outperformed other models with 85.63% accuracy, while Naïve Bayes Bagging was the least accurate at 81.61%
**Schneider et al, 2025^100^**	Apply interpretable machine learning to identify key factors influencing work-related mental health	The authors argue that Decision Tree and Support Vector Machine models achieved over 82% accuracy, with key determinants including work removal and protective measures. High-risk jobs were identified as energy/water operators, legal professionals, and engineers
**Sterling et al, 2022^101^**	Identify and prioritize workplace climate predictors of burnout among primary care physicians in an integrated health system	The authors suggest that identifying workplace climate predictors of exhaustion and depersonalization is crucial for resource allocation to prevent burnout
**Sun et al, 2024^102^**	Investigate the impact of urban integration on migrant workers' mental wellbeing	The study suggests that family urban integration decreases depressive symptoms by 14.6 percentage points, with economic, social, and psychological integration improving well-being
**Sutrisno et al, 2023^103^**	Implement Neural Network to analyze demographic, resilience, COVID-19, and burnout in start-ups	According to the results, 65% of the sample had medium to low resilience, and 61% experienced burnout
**Tang et al, 2023^104^**	Use wearable and survey data to predict burnout risk in shift workers with machine learning	The authors argue that sleep and heart rate are key indicators for predicting burnout risk
**Tavella et al, 2023^105^**	Assess a 34-item burnout measure's ability to distinguish self-identified burnout symptoms	The study suggests that the 34-item measure can differentiate burnout, highlighting exhaustion, cognitive dysfunction, lack of pleasure, and self-criticism as key items
**Van Zyl-Cillié et al, 2024^106^**	Identify factors predicting nurse burnout and emotional exhaustion	According to the results, Gradient Booster classifier achieved the highest accuracy for predicting burnout and emotional exhaustion, with fatigue as the strongest predictor
**Vincent et al, 2021^107^**	Build a model to classify IT employees as depressed or nondepressed	The authors argue that Deep Multilayer Perception with backpropagation outperformed other models for effective classification
**Wang et al, 2021^108^**	Predict medical workers' mental health using machine learning and 32 factors	According to the results, the proposed model achieved 92.55% prediction accuracy, surpassing existing algorithms
**Wang et al, 2025^109^**	Predict depression and anxiety among health care workers	The study reported that 28.37% of health care workers experienced depression and 33.52% experienced anxiety, and that both outcomes were predicted by a range of work-related, demographic, and psychological factors, with being female additionally associated with anxiety
**Wu et al, 2021^110^**	Reinvestigate burnout using social media posts	The authors argue that machine learning models predicted burnout using Weibo data, indicating potential for early intervention
**Yao et al, 2022^111^**	Investigate job-related psychological flexibility, coping style, and personality interactions in depression among Chinese physicians	According to the results job-related psychological flexibility, coping styles, and personality types are associated with depression risks in Chinese physicians
**Yoon et al, 2024^112^**	Assess employment status changes' causal effects on suicidal ideation and depressive symptoms	The study reported that the Random Forest model achieved the highest AUC (0.702) and identified increased suicidal ideation and depressive symptoms among workers in nonstandard employment
**Zadem et al, 2024^113^**	Identify work-related predictors of job burnout via machine learning and questionnaires	The study reported that their model categorized burnout into 3 levels with an overall accuracy of 82%
**Zeng et al, 2025^114^**	Combine Job Demand-Resource model with machine learning to identify burnout factors among Chinese nurses	The authors argue that Random Forest is optimal for predicting burnout, with key predictors being psychological distress, organizational support, emotional intelligence, and D-type personality
**Zhang et al, 2023^115^**	Explore predictors of compassion fatigue among psychological hotline counselors	The study suggests that the key predictors of compassion fatigue are meaning in life, self-efficacy, mindfulness, and empathy
**Zhang et al, 2023^116^**	Detect predisposition to severe psychological distress for timely interventions	Forty-seven variables, including 13 sociodemographic and 34 lifestyle-related, were identified as significant predictors of severe psychological distress
**Zhernova et al, 2020^117^**	Predict early burnout prerequisites among employees	The authors argue that burnout was identified in 70% of cases using Random Forest
**Zhou et al, 2022^118^**	Detect determinants of depressive symptoms among health care workers during COVID-19	The study reported that machine learning models consistently ranked self-perceived health status as the strongest predictor, followed by infection worries, frontline work, uncertainty, psychological support, and COVID-19–like symptoms

Abbreviations: AI, artificial intelligence; BMI, body mass index; ECG, electrocardiogram; HCW, health care worker; HRV, heart rate variability; iCBT, internet-based cognitive behavioral therapy; IT, information technology; MHW, mental healthcare workers; ML, machine learning; NLP, natural language processing; PTSD, post-traumatic stress disorder.

This body of research reported psychological distress experienced by health care workers, IT professionals, and other occupational groups, assessing factors like job-related psychological flexibility, coping styles, and personality traits. Some studies reported research aims that included examining the effects of employment status changes, urban integration, or workplace climate in relation to mental health–related outcomes. In addition, several studies described analyses of textual data to observe temporal patterns in emotional well-being, particularly among health care professionals.

The results of the publications primarily concentrated on the performance of predictive machine learning models in assessing mental health issues. Across the included studies, algorithms such as Random Forest, Gradient Boosting, Support Vector Machines, Neural Networks, and other decision tree–based methods were frequently highlighted for their reported accuracy and related performance metrics. In these studies, accuracy generally referred to the proportion of correctly classified cases, and reported values typically ranged from approximately 70%-80% to over 90%, depending on the modeling task and dataset. Accuracy was often presented alongside other performance indicators such as the area under the receiver operating characteristic (ROC) curve (AUC), sensitivity, specificity, and F1-score, which together described the reported predictive performance of the models.

Several studies reported that their models assigned high or notable importance to variables related to work environment—such as burdensome working conditions, job dissatisfaction, increased workload, lack of professional recognition, and limited social support—as well as demographic and health-related factors including age, education, and body mass index.

Several studies applied machine learning models in a COVID-19–related context and reported elevated levels of anxiety, depression, and burnout among frontline health care workers during the pandemic. According to their results, these outcomes were influenced by variables such as extended working hours, age, and professional responsibilities. A small number of studies that used digital biomarkers or wearable devices reported combining machine-learning models with real-time physiological or behavioral measurements, such as sleep or heart-rate indicators. These studies described incorporating these data sources into their modeling approaches and stated that sensor-based measurements were used to support real-time monitoring of employees’ well-being. In addition, some studies analyzing social media content reported observations on changes or patterns in language use related to mental well-being. A few studies also used social media text to examine indicators they considered potentially relevant for workplace well-being or burnout risk assessment.

## 4. Discussion

This review indicates that the use of machine learning techniques to investigate work-related mental health is still in its preliminary stages. Although the number of publications in this area has increased rapidly during the past 5 years, the focus remains primarily within the domain of data science. There is a strong emphasis on identifying optimal algorithms, with less attention given to carefully planned study designs, the robust formation of study populations, or theoretically ambitious research questions from an occupational health perspective. Although there has been a slight increase in alternative approaches rooted in behavioral sciences, epidemiology, medicine, and social sciences in recent years, they remain in a clear minority. Geographically, while this field is experiencing significant growth in Asia, it remains underdeveloped in other regions. This regional distribution is intriguing considering that most of the work-related mental health research is conducted outside of Asia.

In exploring machine learning applications within mental health research, certain dominant trends have emerged. First, the studies primarily target specific occupational groups, utilizing questionnaire data and occasionally incorporating sensor data. Second, the research focuses on prediction tasks or classification using supervised learning methods, with a preference for algorithms such as Random Forest and Neural Networks. Random Forest clearly appeared as the most frequently used algorithm in the reviewed studies. Prior methodological work highlights several strengths that help explain its widespread use: Random Forest offers high accuracy compared with approaches based on individual models, functions effectively on large datasets, and can handle remarkably high numbers of input variables without the need to remove them. In addition, the method enables unbiased error estimation for balancing category errors and assessing variable importance.^119^

Third, the primary focus is on identifying mental disorders and/or symptoms from questionnaire data, particularly depression, followed by burnout, anxiety, and unspecified mental disorders or work stress–related health outcomes. Fourth, several studies were published during the COVID-19 period and focused on health care workers’ mental distress. The prominence of such studies reflects how the pandemic offered a timely context for applying machine learning approaches that were already becoming more widespread, without implying that the methodological developments themselves were driven by the pandemic.

Despite these advancements, the existing research has several limitations. Large population samples and register data are underutilized, with clinical data being particularly scarce. The predominance of cross-sectional study designs hampers the full utilization of machine learning's predictive capabilities. Prospective designs would be essential for clinical studies and are crucial for advancing medical and socio-behavioral understanding of mental health.^120^^,^^121^ In addition, most studies rely solely on structured data (eg, numerical data on demographic variables, questionnaire measures), rarely exploiting machine learning's potential for processing natural language data.

In addition to these methodological limitations, implementing machine learning approaches in workplace settings raises important ethical and governance considerations that extend beyond research feasibility. Screening or predicting mental health risks at work involves sensitive personal data, and practical application would require robust safeguards related to privacy, informed consent, data security, algorithmic fairness, and the potential misuse of predictions by employers.^122^^,^^123^ These issues were not the focus of the included studies, which primarily examined model performance. Clarifying the distinction between methodological development and practical implementation is essential for understanding how machine learning tools could be responsibly integrated into occupational health contexts.^124–126^

Although machine learning models frequently demonstrated satisfactory performance across the included studies, these reported results require cautious interpretation. The predominance of cross-sectional designs, variability in sample sizes, and the limited use of external validation constrain the generalizability of model performance. Overall, while the reviewed studies indicate promising potential for machine learning methods in occupational mental health research, stronger validation procedures and more transparent reporting will be essential to establish the robustness of these models in applied settings.

### 4.1 Implications for future research

The findings of this literature review align with those of Hashmi and Yadav,^10^ who examined the application of machine learning in stress research. Our review suggests that the field is evolving in many respects, with aspects such as representativeness and transparency of datasets requiring further attention. One of the key areas for improvement identified in our review is clarity. Specifically, several studies could benefit from a clearer connection to existing literature on mental health at work, as well as more defined objectives regarding occupational mental health.

A possible explanation for these findings is that the use of machine learning in this field is currently primarily led by data scientists. Researchers specializing in occupational and mental health have not yet widely integrated machine learning methods into their study approaches and research tools, often relying on traditional research approaches. Consequently, existing machine learning–based studies in occupational mental health may not yet fully align with the research questions and approaches employed by occupational health scientists, social epidemiologists, or behavioral scientists, suggesting potential opportunities for further collaborative contributions to the field.

However, the findings of this analysis also highlight the significant potential of machine learning methods in advancing the study of occupational mental health. Enhanced predictive capabilities are one of the most promising aspects, with models such as Decision Trees and Gradient Boosting demonstrating robust performance in anticipating mental health outcomes. Machine learning also offers the opportunity to integrate diverse data types into research, thereby enriching the understanding of mental health influences. Studies have already utilized data sources such as heart rate variability, sociodemographic factors, and social media content. By incorporating additional data types, like sensor data and text data, researchers can develop a more comprehensive overview of the factors contributing to mental health in different contexts of labor market.

Moreover, machine learning can automate the screening process for mental disorders, enabling early detection and timely referral to professional assistance—an approach that is particularly beneficial for high-risk groups, including frontline health care workers. Furthermore, machine learning approaches have the potential to facilitate the development of novel theoretical frameworks for the study of work and mental health in the context of evolving labor markets and technological shifts, which are known to transform the nature of work and impact work ability.

Overall, machine learning methods still appear to remain underutilized in the study of occupational mental health, highlighting the need for more ambitious research efforts. Studies from other fields have demonstrated how machine learning can analyze medical records containing both structured information and natural language to identify risk factors and individuals at risk, potentially providing new preventive tools for health care providers.^127^^,^^128^ These approaches could be further developed and applied to examine mental health in the workplace and other aspects of occupational health.

### 4.2 Strengths and limitations

This study presented several strengths, marking it as a pioneering effort in evaluating the use of machine learning methods in workplace mental health research through a comprehensive literature search. By systematically analyzing publications across multiple databases, the study ensured a robust and wide-ranging assessment of existing literature. It offers valuable information on the approaches, study designs, methodologies, and populations utilized in the field. Additionally, the study identifies the key strengths and limitations in the field, providing a foundational understanding and setting the stage for future interdisciplinary collaboration to enhance the potential of machine learning in advancing occupational mental health research.

However, it is noteworthy that this review did not identify any publications utilizing large language models (LLMs) such as ChatGPT, introduced in late 2022. Importantly, our search strategy did not include specific terms related to LLMs or transformer-based architectures (eg, “large language model,” “transformer,” “ChatGPT,” “BERT”), as the search was designed around broader machine learning terminology. As a result, the absence of LLM-based studies in our findings possibly reflects the scope of the search strategy rather than their absence in the wider literature. While LLMs offer promising opportunities for applications in workplace mental health research, their use is also constrained by data security concerns and ethical issues such as plagiarism and biased content.^129^ Future reviews should explicitly incorporate LLM-related search terms to better capture emerging developments in this area.

## 5. Conclusions

Machine learning techniques hold significant potential for advancing research on work-related mental health, yet their use remains limited outside data science–driven method development. Greater interdisciplinary collaboration, particularly with epidemiology, medicine, behavioral sciences, and the social sciences, could broaden the field and enable the integration of richer data sources, including clinical data and natural language data.

To fully leverage the potential of machine learning in this field, research should clearly distinguish between methodological contributions and studies aimed at generating substantive insights into workplace mental health. Prospective, longitudinal designs with separate predictor and outcome datasets would better support robust prediction. Also, NLP offers promise for capturing emotional well-being.

Overall, progress will require ambitious, high-quality interdisciplinary studies that apply innovative techniques from other domains. Such efforts can help create effective preventive tools for health care providers and establish machine learning as a meaningful contributor to understanding and improving mental health in the workplace.

## Supplementary Material

uiag014_Supplemental_Files

## Data Availability

The data for this study were extracted from published scientific literature. All data are presented in both the article and its Supplementary material.
