# Supplementary material for: Machine learning in the analysis of mental health at work: a scoping review
Source: J Occup Health. 2026 Mar 9;68(1):uiag014. doi: 10.1093/joccuh/uiag014 (PMC13219837; doi:10.1093/joccuh/uiag014)
Supplement: uiag014_Supplemental_Files [file uiag014_supplemental_files.zip › Supplementary_file_uiag014.docx]

**Supplementary Table 1** Search strategy overview.

| **Platform** | **Search date and hits** | **Databases searched** | **Search terms** |
| --- | --- | --- | --- |
| EBSCOhost | Initial search on 27 June 2023: 976 hits  Updated search on 15 September 2025: 1424 hits | Academic Search Complete; CINAHL; MEDLINE; SocINDEX with Full Text | ( “mental health” OR “occupational stress” OR “work stress” OR “work-related stress” OR “psychological distress” OR “psychiatric disorder*” OR “mental disorder” OR depression OR “work-related depression” OR burnout OR “occupational burnout” OR anxiet* ) AND ( “occupational health” OR “occupational health service*” OR “occupational health care” OR “work life” OR “working population” OR workplace* OR employe* OR worker ) AND ( “artificial intelligence” OR “machine learning” OR “natural language process*” OR “risk algorithm*” OR “data mining” ) |
| Scopus | Initial search on 27 June 2023: 844 hits  Updated search on 15 September 2025: 1853 hits |  | (TITLE-ABS-KEY("mental health" OR "occupational stress" OR "work stress" OR "work-related stress" OR "psychological distress" OR "psychiatric disorder*" OR "mental disorder" OR depression OR "work-related depression" OR burnout OR "occupational burnout" OR anxiet*) AND TITLE-ABS-KEY("occupational health" OR "occupational health service*" OR "occupational health care" OR "work life" OR "working population" OR workplace* OR employe* OR worker*) AND TITLE-ABS-KEY("artificial intelligence" OR "machine learning" OR "natural language process*" OR "risk algorithm*" OR "data mining")) |
| ProQuest | Initial search on 27 June 2023: 100 hits  Updated search on 15 September 2025: 159 hits | Education Collection; International Bibliography of the Social Sciences (IBSS); Social Science Database; Sociology Collection | noft("mental health" OR "occupational stress" OR "work stress" OR "work-related stress" OR "psychological distress" OR "psychiatric disorder*" OR "mental disorder" OR depression OR "work-related depression" OR burnout OR "occupational burnout" OR anxiet*) AND noft("occupational health" OR "occupational health service*" OR "occupational health care" OR "work life" OR "working population" OR workplace* OR employe* OR worker*) AND noft("artificial intelligence" OR "machine learning" OR "natural language process*" OR "risk algorithm*" OR "data mining") |
| Web of Science Core Collection | Initial search on 27 June 2023: 559 hits  Updated search on 15 September 2025: 1041 hits | Science Citation Index Expanded (SCI‑EXPANDED) — 1945‑present; Social Sciences Citation Index (SSCI) — 1956‑present; Arts & Humanities Citation Index (AHCI) — 1975‑present; Conference Proceedings Citation Index – Science (CPCI‑S) — 1990‑present; Conference Proceedings Citation Index – Social Science & Humanities (CPCI‑SSH) — 1990‑present; Book Citation Index – Science (BKCI‑S) — 2005‑present; Book Citation Index – Social Sciences & Humanities (BKCI‑SSH) — 2005‑present; Emerging Sources Citation Index (ESCI) — 2015‑present | Search: ((TS=(“artificial intelligence” OR “machine learning” OR “natural language processing” OR “risk algorithm” OR “data mining”)) AND TS=(“occupational health” OR “occupational health service*” OR “occupational health care” OR “work life” OR “working population” OR workplace* OR employe* OR worker*)) AND TS=(“mental health” OR “occupational stress” OR “work stress” OR “work-related stress” OR “psychological distress” OR “psychiatric disorder” OR “mental disorder” OR depression OR “work-related depression” OR burnout OR “occupational burnout” OR anxiety) |
| APA PsycInfo | Initial search on 27 June 2023: 136 hits  Updated search on 15 September 2025: 285 hits |  | 1. mental health.mp. OR exp Mental Health/  2. occupational stress.mp. OR exp Occupational Stress/  3. work stress.mp.  4. work-related stress.mp.  5. psychological distress.mp.  6. psychiatric disorder*.mp.  7. exp Mental Disorders/ OR mental disorder*.mp.  8. depression*.mp.  9. work-related depression.mp.  10. exp Burnout/ OR burnout.mp.  11. occupational burnout.mp. OR exp Occupational Stress/  12. exp Anxiety/ OR anxiet*.mp.  13. 1 OR 2 OR 3 OR 4 OR 5 OR 6 OR 7 OR 8 OR 9 OR 10 OR 11 OR 12  14.mp. OR exp Artificial Intelligence/  24. machine learning.mp. OR exp Machine Learning/  25. exp Natural Language Processing/ OR natural language process*.mp.  26. risk algorithm*.mp.  27. data mining.mp. OR exp Data Mining/  28. 23 OR 24 OR 25 OR 26 OR 27  29. 13 AND 22 AND 28  30. limit 29 to "remove medline records" |
| IEEE Xplore Digital Library | Initial search on 27 June 2023: 572 hits  Updated search on 15 September 2025: 967 hits |  | ("All Metadata":“work stress” OR "All Metadata":“work-related stress” OR "All Metadata":“psychological distress” OR "All Metadata":“psychiatric disorder*” OR "All Metadata":“mental disorder” OR "All Metadata":depression OR "All Metadata":“work-related depression” OR "All Metadata":burnout OR "All Metadata":“occupational burnout” OR "All Metadata":anxiet*) AND ("All Metadata":“occupational health” OR "All Metadata":“occupational health service*” OR "All Metadata":“occupational health care” OR "All Metadata":“work life” OR "All Metadata":“working population” OR "All Metadata":workplace* OR "All Metadata":employe* OR "All Metadata":worker*) AND ("All Metadata":“artificial intelligence” OR "All Metadata":“machine learning” OR "All Metadata":“natural language process*” OR "All Metadata":“risk algorithm*” OR "All Metadata":“data mining”) |
| ACM Digital Library | Initial search on 27 June 2023: 89 hits  Updated search on 18 September 2025: 54 hits |  | [[Abstract: "mental health"] OR [Abstract: "occupational stress"] OR [Abstract: "work stress"] OR [Abstract: "work-related stress"] OR [Abstract: "psychological distress"] OR [Abstract: "psychiatric disorder"] OR [Abstract: "mental disorder"] OR [Abstract: depression] OR [Abstract: "work-related depression"] OR [Abstract: burnout] OR [Abstract: "occupational burnout"] OR [Abstract: anxiety]] AND [[Abstract: "occupational health"] OR [Abstract: "occupational health service"] OR [Abstract: "occupational health care"] OR [Abstract: "work life"] OR [Abstract: "working population"] OR [Abstract: workplace] OR [Abstract: employee] OR [Abstract: worker]] AND [[Abstract: "artificial intelligence"] OR [Abstract: "machine learning"] OR [Abstract: "natural language processing"] OR [Abstract: "risk algorithm"] OR [Abstract: "data mining"]] |

**Supplementary Table 2** Data extraction template.

| **Category** | **Question** | **Options** |
| --- | --- | --- |
| General information | Publication title | [Open field] |
|  | Publication year | [Open field] |
|  | Country in which the study conducted | 1. USA 2. Asia excluding China 3. China 4. Europe 5. The Americas excluding USA 6. Africa 7. Oceania 8. Multi-region   9. Not reported |
| Methods and study design | Aim of study | [Open field] |
|  | Study design | 1. Randomised controlled trial 2. Non-randomised experimental study 3. Cross sectional study 4. Review 5. Prevalence study at population level 6. Individual level social media analysis 7. Societal level social media analysis 8. Prospective study 9. Other [Open field] |
|  | Main discipline | 1. Computer science 2. Epidemiology 3. Medical science incl. psychiatry 4. Behavioral sciences 5. Social sciences 6. Other [Open field] |
|  | Type of data | - 1. Open source survey data   2. Clinical data   3. National register data   4. Online questionnaire   5. Workplace questionnaire   6. Sensor data   7. Social media   8. Other qualitative data   9. Other [Open field] |
|  | Used any unstructured text? | 1. Yes 2. No |
|  | Methods | 1. NLP 2. Data mining 3. Clustering 4. Prediction 5. Classification 6. Other [Open field] |
|  | Algorithms | [Open field] |
|  | Supervised or unsupervised methods | 1. Supervised 2. Unsupervised |
|  | Outcomes | 1. Depression 2. Anxiety 3. Burnout 4. Work stress 5. Psychological distress 6. Other [Open field] |
|  | Type of outcome | 1. Clinical or register based 2. Self-reported |
| Participants | Population description | 1. Organizational group 2. Occupational group 3. General population sample 4. Patients or employees with mental health diagnosis 5. Unspecific online recruitment 6. Other [Open field] |
|  | Total number of participants | [Open field] |
| Results and application | Medical application | 1. Diagnosis 2. Prognosis 3. Epidemiology 4. Treatment 5. None [Open field] |
|  | Main results | [Open field] |
